# These may not be the courses you are seeking: a systematic review of open online courses in health professions education

**DOI:** 10.1186/s12909-019-1774-9

**Published:** 2019-09-14

**Authors:** Michael Rowe, Christian R. Osadnik, Shane Pritchard, Stephen Maloney

**Affiliations:** 1 Department of Physiotherapy, Faculty of Community and Health Sciences, University of the Western Cape, Cape Town, South Africa; 2 Department of Physiotherapy, School of Primary and Allied Health Care, Monash University, Frankston, Australia

**Keywords:** Open online courses, Health professions education, Systematic review

## Abstract

**Introduction:**

Open Online Courses (OOCs) are increasingly presented as a possible solution to the many challenges of higher education. However, there is currently little evidence available to support decisions around the use of OOCs in health professions education. The aim of this systematic review was to summarise the available evidence describing the features of OOCs in health professions education and to analyse their utility for decision-making using a self-developed framework consisting of point scores around effectiveness, learner experiences, feasibility, pedagogy and economics.

**Methods:**

Electronic searches of PubMed, Medline, Embase, PsychInfo and CINAHL were made up to April 2019 using keywords related to OOC variants and health professions. We accepted any type of full text English publication with no exclusions made on the basis of study quality. Data were extracted using a custom-developed, a priori critical analysis framework comprising themes relating to effectiveness, economics, pedagogy, acceptability and learner experience.

**Results:**

54 articles were included in the review and 46 were of the lowest levels of evidence, and most were offered by institutions based in the United States (*n* = 11) and United Kingdom (*n* = 6). Most studies provided insufficient course detail to make any confident claims about participant learning, although studies published from 2016 were more likely to include information around course aims and participant evaluation. In terms of the five categories identified for analysis, few studies provided sufficiently robust evidence to be used in formal decision making in undergraduate or postgraduate curricula.

**Conclusion:**

This review highlights a poor state of evidence to support or refute claims regarding the effectiveness of OOCs in health professions education. Health professions educators interested in developing courses of this nature should adopt a critical and cautious position regarding their adoption.

**Electronic supplementary material:**

The online version of this article (10.1186/s12909-019-1774-9) contains supplementary material, which is available to authorized users.

## Background

Open Online Courses (OOCs), including Massive Open Online Courses (MOOCs), have been characterised as “the next evolution of networked learning” [1] and identified as a platform that may expand access to higher education and support innovative teaching practices. Coined in 2008, MOOCs refer to online courses offered by institutions that attract thousands of participants, partly due to the fact that they are “open”, which usually refers to the fact that they are not credit-bearing and therefore free to anyone with an internet connection. While formal research in this emerging field is limited, many supporters of the format have embraced its implementation with enthusiasm [2]. There has been a dramatic increase in the development and implementation of MOOCs across many aspects of higher education and, more recently, within health professions education [3].

Few studies have demonstrated significant benefits of OOCs on either student learning, professional workforce shortages, or the need to disrupt more “traditional” approaches to teaching and learning. The lack of evidence in the field of health professions education has not, however, diminished the enthusiasm with which they are discussed [4, 5]. Mehta and colleagues (2013) [5] suggest that “no longer will a limited number of medical schools or faculty constrain our ability to educate medical students” and that “learning communities will form naturally, and students will need to take ownership of their education”. However, this also articulates a divide between pedagogical *vision* and professions founded upon *evidence-based* principles.

To date, the most comprehensive review of MOOCs in health professions education has been by Liyanagunawardena and colleagues (2014) [3]. This review provided detailed overviews of the courses themselves but, importantly, did not appraise and synthesise the evidence regarding their *effectiveness*. Their conclusion, that MOOCs have the potential to make an important contribution to health professions education, was therefore not founded upon evidence. This lack of evidence is not limited to studies of OOCs in health professions and medical education. Critically reviewed literature is also scarce in the domain of OOCs in the more general higher education literature [6]. This weak foundation poses significant issues for academic institutions responsible for the design and implementation of evidence-based models of health professions education, and who are considering the large-scale adoption of MOOCs in their curricula.

This does not mean OOCs lack the potential to disrupt health professions education. There is evidence that they may introduce broader social connections, opportunities for enhanced collaboration, and exposure to many different perspectives, all of which change the educational space in ways that may improve student learning. The original MOOCs were informed by emergent theories of knowledge and learning, such as connectivism, and supported the development of socially-negotiated and relationally-constructed knowledge, as well as moving the teacher towards the periphery of the learning interaction [7]. These environments may facilitate a type of learning that is self-organised, collaborative, and open, where the learner is at the centre of the process. The networked nature of the course leads to a high number of interactions between people and resources, where learners organise and determine the process and to some extent the outcomes, making the course relatively unpredictable [7]. It may be that this disruptive innovation has the potential to significantly change how we think about learning in the twenty-first century [8] or it may simply be a “good thing to think with” [9]. It is presently difficult to say with confidence whether MOOCs in health professions education enhance student learning or not.

This systematic review therefore aimed to 1) summarise the available evidence describing the use of OOCs in health professions education; 2) describe the features of these courses; and 3) determine their effectiveness against performance outcomes of relevance to health professions education providers.

## Methods

The protocol for this review was registered on PROSPERO in July 2016 (#CRD42016042421). Ethics approval was deemed unnecessary for this study as it was a systematic review of the literature. Electronic searches of PubMed, Medline, Embase, PsychInfo and CINAHL databases were conducted from inception to April 2019 to identify relevant publications in the field of OOCs in health professions education. Each database was searched using the following terms: ‘massive open online course’ OR ‘MOOC’ OR ‘open online course’ OR ‘OOC’ OR ‘distributed online collaborative course’ OR ‘DOCC’ OR ‘small private online course’ OR ‘SPOC’, without any restrictions. The last two terms were included due to their relatively broad context and potential to identify relevant studies (despite not being truly ‘open’ in nature). The intervention was defined as any OOC that was designed to address an aspect of health considered relevant to the scope of practice of health professional students. Courses targeting undergraduate or postgraduate training were deemed appropriate for inclusion.

As we expected the search to yield a wide variety of studies, no exclusions were made on the basis of study type. Studies must have been published in full text, English language and targeted towards any of the following health professionals: medicine, physiotherapy, occupational therapy, nursing, radiology, speech and language therapy, dietetics, public health, dentistry and psychology. Grey literature was identified via Google Scholar using the same search terms as per the database searches, with any literature included if it was identified from the first three pages of the google search. Reference lists of included studies were hand-searched.

Study selection and data extraction were undertaken by two members of the research team, with random accuracy checks provided by another team member. Discrepancies were resolved by a third member of the team (when relevant) to derive consensus. We developed and piloted a standardised data extraction form to identify the key study characteristics (year and location of publication), study type (methodology), participant characteristics, key outcomes using a self-developed framework (described further), and quality appraisal. Assessment of risk of bias of included studies was undertaken using instruments specific to individual study designs. This approach limits the ability to pool judgments across studies but enables greater depth of evaluation within studies, in keeping with the focus of this review. Randomised controlled trials were evaluated using the Cochrane Risk of Bias tool; reviews evaluated via the AMSTAR checklist; other study types were evaluated using the suite of The Joanna Briggs Institute quality appraisal instruments for cohort studies, pre/post test studies and commentaries/expert opinion. The ‘level of evidence’ was defined for all studies according to the extended version of the Australian National Health and Medical Research Council (NHMRC) hierarchy for intervention studies [10]. This hierarchy is the reference standard for appraising levels of evidence for health technology assessment in Australia and was developed following an extensive four-year pilot process involving a combination of evidence, theory and consultation, informed by existing tool such as those used by the National Institute for Clinical Excellence (adapted from the Scottish Intercollegiate Guidelines Network) [11], the National Health Service Centre for Reviews and Dissemination [12] and the Centre for Evidence Based Medicine (CEBM) hierarchy [13]. Individual studies are rated with a score ranging from I (systematic reviews of randomised controlled trials) to IV (case series with either post-test or pre-test/post-test outcomes), with higher scores equating to higher levels of evidence. Commentary or expert opinion papers do not feature on this scale, so were attributed a score of ‘V’ (lowest form of evidence). No studies were excluded from the review on the basis of study quality.

Given the relative infancy of research in this field, data were not anticipated to be suitable for inclusion in a meta-analysis of primary and secondary outcomes. Data were therefore analysed using a mixed-methods approach of quantitative synthesis (incorporating descriptive summary statistics) and narrative summary of relevant data regarding the impact of OOCs in health professions education. In order for data to be permissible, findings needed to be clearly interpretable via either quantitative (e.g. summary statistics, count data) or qualitative means (e.g. user experience statements). In order to optimise the relevance of OOC research in the field of health professions education, data needed to be evaluated against metrics of importance to education administrators and performance outcomes. We reviewed the available literature to identify suitable tools for the purpose of such directed reporting but failed to identify any that contained the requisite detail for this study. Review findings were therefore summarised using a user-defined OOC evaluation framework, defined a priori for this review, that comprised five key outcome ‘pillars’, as follows:

1. Effectiveness (primary outcome): i.e. did the OOC increase learner knowledge?
2. Learner perceptions (opinions / attitudes): i.e. was the OOC enjoyable or rewarding?
3. Acceptability (feasibility / usability): i.e. how well could learners engage with the OOC?
4. Pedagogy: i.e. was the OOC based upon a stated educational framework or theory?
5. Economics: i.e. was the OOC evaluated against a measure of cost and/or value?

Data from each study were mapped against each pillar to derive five quantitative point estimates that reflected the total number of studies providing admissible data. These data were summarised as a percentage of the total number of included studies and represented visually via radar graph using Microsoft Excel. Qualitative data such as participant testimonies or user feedback was considered requisite evidence to satisfy the meeting of any OOC pillar.

## Results

### Aim 1 (overview of included studies of OOCs in health professions education)

**Fig. 1:**
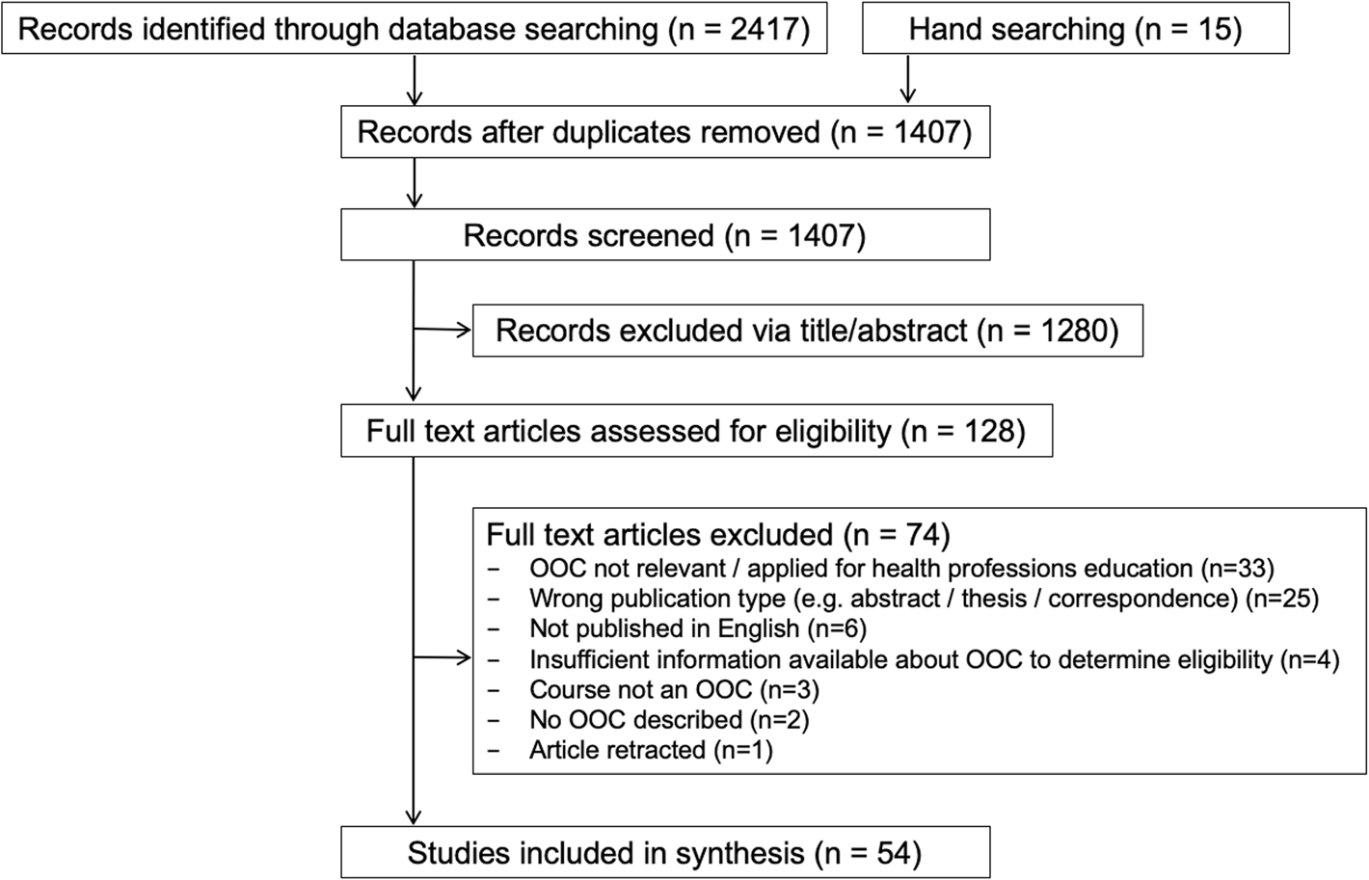
(PRISMA flowchart)

The electronic database search yielded 2417 records and hand-searching retrieved an additional 15 studies. After de-duplication and removal of records based on title and abstracts, we screened 128 full-text articles against the inclusion criteria, resulting in 54 articles being included in the review (Fig. 1)

Detailed information regarding the characteristics of included articles is presented in Table 1. Most included papers were of a narrative / opinion (*n* = 24) or descriptive / case series (*n* = 22) design, meaning 46 of the 54 included articles were deemed to be of the lowest levels of evidence (levels IV / V) according to the NHMRC hierarchy. One randomised controlled trial (RCT) and two cohort/case control studies were included. Four review articles were included, however none were systematic reviews of RCTs (level I evidence). The RCT was deemed to be at high risk of bias due to lack of blinding of participants to knowledge of group allocation, which may have affected self-reported outcome data. Complete details regarding quality appraisals of individual studies are provided in the Additional file 1: Table S1, Additional file 2: Table S2, Additional file 3: Table S3, Additional file 4: Table S4, Additional file 5: Table S5, Additional file 6: Table S6.

**Table 1:** Characteristics of included studies

Study ID	Design	Health profession(s)	N	Course country	Course duration	Level of evidence
Bellack 2013 [14]	Narrative / opinion	Nursing	Unreported	Unreported	Unreported	V
Billings 2014 [15]	Narrative / opinion	Nursing	Unreported	USA	Unreported	V
Coughlan 2015 [16]	Narrative / opinion	Any (mainly physiotherapy and psychiatry)	Unreported	UK	Unreported	V
Davies 2013 [17]	Narrative / opinion	Medicine	Unreported	Unreported	Unreported	V
DeSilets 2013 [18]	Narrative / opinion	Any (mainly medicine and nursing)	Unreported	Various	Unreported	V
Evans 2017 [19]	Descriptive / case series	Various	7082	Various	6 wks	IV
Frank 2016 [20]	Cohort / case control	Medicine	202	USA	Unclear	III-3
Fricton 2015 [21]	Descriptive / case series	Any (incl. Non health-professionals)	23,650	USA	18 wks	IV
Geissler 2015 [22]	Narrative / opinion	Nutrition and dietetics	Unreported	UK	Unreported	V
Goldberg 2017 [23]	Narrative / opinion	Any health professions	Unreported	Unreported	Unreported	V
Gooding 2013 [24]	Descriptive / case series	Public health	Unreported	USA	Unreported	IV
Harder 2013 [4]	Narrative / opinion	Medicine	Unreported	USA	Unreported	V
Harvey 2014 [25]	Descriptive / case series	Physiotherapy	3523	UK	5 wks	IV
Harvey 2017 [26]	Descriptive / case series	Physiotherapy	13,509	Various	5 wks	IV
Heller 2014 [27]	Narrative / opinion	Any health professions	Unreported	Unreported	Unreported	V
Henningsohn 2017 [28]	Narrative / opinion	Medicine	4925	Various		V
Hoedebecke 2018 [29]	Descriptive / case series	Medicine	40	Unclear	Unreported	IV
Hossain 2015 [30]	RCT	Physiotherapy	48	UK	5 wks	II
Hoy 2014 [31]	Narrative / opinion	Medicine	Unreported	Unreported	Unreported	V
Inácio 2015 [32]	Review	Pharmacy	Unreported	Finland / Portugal	Unreported	I
Jacquet 2018 [33]	Descriptive / case series	Unclear	5935	USA	Unreported	IV
Jia 2019 [34]	Cohort / case control	Nursing	4016	China	16 wks	III-3
Juanes 2015 [35]	Narrative / opinion	Any health professions	Unreported	Unreported	Unreported	V
Kearney 2016 [36]	Narrative / opinion	Dentistry	Unreported	Unreported	Unreported	V
King 2014 [37]	Narrative / opinion	Any health professions	10,000	Australia	11 wks	V
King 2015 [38]	Descriptive / case series	Any (incl. Carers for people with dementia)	Unreported	Australia	Unclear	IV
Kononowicz 2015 [39]	Descriptive / case series	Behavioural Medicine	19,236	Sweden	5 wks	IV
Lan 2019 [40]	Descriptive / case series	Dentistry	7608	China	5 wks	IV
Liyanagunawardena 2014 [3]	Review	Any health professions	Unreported	Unreported	Unreported	I
Liyanagunawardena 2018 [41]	Narrative / opinion	Unreported	Unreported	Unreported	Unreported	V
Lunde 2018 [42]	Descriptive / case series	Medicine and Nursing	Unreported	Unreported	Unreported	IV
Magana 2018a [43]	Descriptive / case series	Various	35,968	Mexico	Unreported	IV
Magana 2018b [44]	Descriptive / case series	Various	19,563	Mexico	40 h	IV
Masters 2011 [45]	Narrative / opinion	Medicine	Unreported	Unreported	Unreported	V
Maxwell 2018 [46]	Narrative / opinion	Various	Unreported	Various	Unreported	V
McCartney 2015 [47]	Narrative / opinion	Nursing	Unreported	Unreported	Unreported	V
Medina 2017 [48]	Descriptive / case series	Medicine and Nursing	1169	Various	6 wks	IV
Milligan 2014 [49]	Descriptive / case series	Any health professions	22,000	USA	4 mths	IV
Perez-Moreno 2018 [50]	Descriptive / case series	Medicine and Pharmacy	2148	Spain	4 mths	IV
Power 2015 [51]	Narrative / opinion	Nursing and Midwifery	Unreported	Unreported	Unreported	V
Roberts 2014 [52]	Narrative / opinion	Medicine	Unreported	USA	Unclear	V
Robinson 2016 [53]	Descriptive / case series	Medicine	40	USA	5 wks	IV
Rowe 2016 [54]	Qualitative	Physiotherapy	8	South Africa	6 wks	IV
Sitzman 2016 [55]	Descriptive / case series	Nursing	714	USA	4 wks	IV
Skiba 2013 [56]	Narrative / opinion	Nursing	Unreported	Unreported	Unreported	V
Sneddon 2018 [57]	Descriptive / case series	Various	32,944	Various	6 wks	IV
Stokes 2015 [58]	Descriptive / case series	Dentistry (potential enrolees)	4224	UK	6 wks	IV
Subhi 2014 [59]	Review	Medicine	Unreported	Unreported	Unreported	I
Swinnerton 2017 [60]	Descriptive / case series	Medicine	18,382	UK	3 wks	IV
Szpunar 2013 [61]	Narrative / opinion	Psychology (plus economics and classics)	Unclear	Unclear	1X1-hr lecture	V
Takooshian 2016 [62]	Narrative / opinion	Psychology	Unreported	Unreported	Unreported	V
Unknown 2015 [63]	Narrative / opinion	Unclear	Unclear	USA	Unclear	V
Wan 2016 [64]	Descriptive / case series	Pharmacy	407	Taiwan	4 wks	IV
Zhao 2018 [65]	Review	Medicine	12,197	Various	Unclear	V

*Unreported* not applicable, *RCT* randomised controlled trial, *UK* United Kingdom, *USA* United States of America, NHMRC levels of evidence: *II* a randomised controlled trial, *III-2* a comparative study, *IV* case series or cross-sectional study, *V* expert opinion or other

### Aim 2 (features of OOCs in this study)

No single health profession was overtly over or under-represented with a spread of courses offered across medical, nursing and the allied health professions. Most courses were delivered by academic centres from either the United States of America (*n* = 11), the United Kingdom (*n* = 6) or Australia and China (*n* = 2). The number of participants enrolled in OOCs ranged from as low as 8 (who were participating in a qualitative study) to as high as 35,968. OOCs were reported to have been offered for durations ranging from a single session of one hour to 18 weeks. Some uncertainty existed regarding the precise course duration for some studies (see Table 1 for additional detail).

Of the 36 studies that provided sufficient detail to describe the online course, 32 defined the aim(s) of the OOC. Most were developed with the intent of improving participants’ knowledge and 15 studies reported outcome data related to this aim.

Sixteen studies defined the methods of assessment for evaluating the OOC. Many articles incorporated online quizzes to assess the extent of knowledge acquisition, either after an individual module or upon conclusion of the OOC. Two of these studies reported the use of baseline testing. Two studies required the submission of a written essay to evaluate the impact of the course [15, 57], one of which was peer reviewed [57].

Most OOCs involved at least one element of participant ‘interaction’ although more recent articles included 3–5 different interactive elements. These included embedded video lectures with interactive revision questions, online lessons, discussion forums for peer engagement, or formative quizzes (e.g. multiple choice questions) that were either mandatory or voluntary. Most OOCs presented course materials using existing platforms such as Coursera, Udemy, EdX, and Canvas.

### Aim 3 (evaluation of the effectiveness of OOCs for health professions education)

**Fig. 2:**
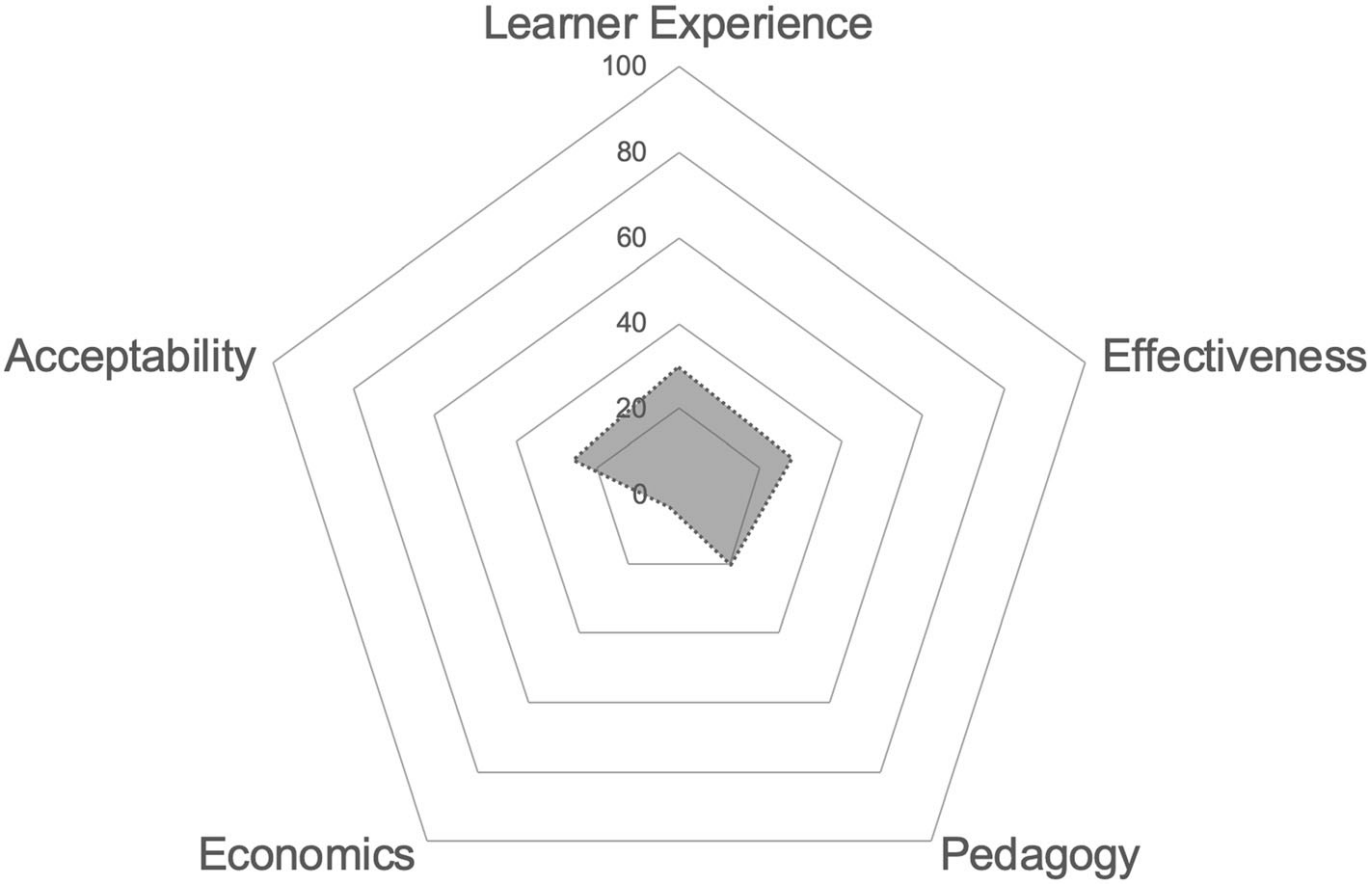
Numbers in the figure refer to the percentage of included studies for each pillar in the evaluation framework

As anticipated, data were not suitable for formal meta-analysis. The very low percentage of studies that reported against any of the core outcomes (indicated by the small area of shading relative to the total graph region in Fig. 2 below) demonstrates that the evaluation of OOCs against outcomes of importance to health professions educators was rare. This was particularly evident across the ‘economic’ and ‘pedagogical’ pillars of our outcome framework.

### Effectiveness

Twenty-three studies presented participant self-reported data concerning changes in knowledge and or behaviours of the learners after completion of the MOOC. The following descriptions are presented as examples of the ways in which articles report on the effectiveness of the courses with respect to achieving the stated aims. One paper provided comparative data with self-directed learning, revealing no differences between groups for either knowledge or perceived confidence in patient management. Another reported that 85% of its health professional learner participants believed that it changed the care of their patients (*n* = 300). Another reported that 93% of its participants believed the course had changed their lives (*n* = 516). Two studies [20, 34] attempted to use controls to determine differences in outcomes between respondents who had used MOOCs in isolation vs respondents who had used MOOCs in addition to “traditional” courses. One qualitative study [54] attempted to map students’ responses from focus group discussions to Herrington’s authentic learning framework [66] as a way to demonstrate the achievement of learning outcomes related to the development of graduate attributes.

Only one included study was a randomised controlled trial that directly compared the effect of a MOOC to an alternative model of education. This study by Hossain et al. (2015) [30] compared the delivery of a 5-week online learning module on spinal cord injuries via either a weekly guided MOOC with Facebook interactive discussions to a conventional self-paced module in a small sample of undergraduate physiotherapy students from Bangladesh and evaluated its effectiveness in improving knowledge, confidence and/or satisfaction. The study failed to demonstrate any significant favorable effects of the MOOC model of education on these outcomes. Students also reported some positive aspects of the MOOC relating to the unique opportunities it afforded to interacting with students from other countries. While this study does offer some insight into the use of MOOCs in health professions education in general, the findings should be interpreted with caution, especially considering the high risk of bias as a result of the lack of blinding.

### Learner experience (attitudes of health professionals toward their learning)

Seven studies reported on outcome measures relating to the learner experience of participating in the MOOC. The most common measure was participant satisfaction with twelve studies reporting overwhelmingly positive experiences of participation in MOOCs. However one study [30] reported the participants were neutral in their assessment of satisfaction (Likert Scale score of 0.0 (95% CI − 1.1 to 1.2), and another [21] reported that only 56% of learners were satisfied with the quality of the course discussion forums.

One study [39] provided strong agreement from participants for the helpfulness of a virtual patient experience. One study [21] included qualitative comments from participants, reporting that the course helped with self-discovery, and expanded their view of the world. Whereas another [25] reported that they found the course provided an opportunity engage with other health professionals and health professional students from around the globe.

### Acceptability (feasibility / usability)

Few studies reported participant feedback on the acceptability (feasibility or usability) of the OOC format. This item was focused on the self-reported ability of the learner to effectively engage with the course learning materials and methods. Findings included studies reporting the course being ‘too technical’ (*n* = 1), trying to be too many things to too many people (n = 1), an excess of interactive screens (n = 1), technical problems for approximately 16% of participants such as broken sessions and issues concerning internet connectivity (n = 1), taking too much time (n = 1) and an excessive number of discussion posts and threads (n = 1). In addition, one study [60] found participants believed the course was a valuable supplement to the existing “traditional” course but that it should not be used as a replacement.

### Pedagogy

While three studies [25, 30, 39] specifically described the included courses as xMOOCs, most of the descriptive studies included in this review described couse features that would fit into an xMOOC-type design. These were characterised by features such as embedded video lectures, assigned reading texts, answering multiple choice questions, and participating in forum discussions. Another study [54] reported on the course design as being informed by cMOOCs and described the use of authentic learning as a pedagogical framework for the course structure [54]. Finally, one study [64] reported on the use of the ADDIE model of instructional design (Analysis, Design, Development, Implementation, and Evaluation) in order to develop the course. No other articles reported on the development process of any courses.

### Economics

While two articles included information related to the expense of course development (50,000 Euros and 10,000–50,000 dollars) [28, 46], no studies reported outcome measures relating to either a simple cost or value analysis, or comparative costs in the form of cost-benefit or cost-effectiveness analysis. We looked for evidence across the full spectrum of cost and value analyses, including cost-analyses (where outcomes are not considered), and breakeven analyses, and comparative approaches such as cost-minimisation analyses (where the outcomes are assumed equal), cost-benefit analyses (where costs and effects are considered in monetary units), and cost-effectiveness analyses (where outcomes are retained in natural units, such as measures of learning) [67, 68].

## Discussion

This is the first review to systematically investigate the published literature regarding the use and efficacy of OOCs in the field of health professions education. The most prominent outcome from this review is the striking imbalance between the state of anecdotal buoyant enthusiasm for their use in education practice compared to the robustness of the evidence regarding their effectiveness – only 54 papers were deemed eligible for inclusion, with 46 of these defined as low level evidence according to the NHMRC hierarcy we used. This is a significant concern. While some may argue that progress need not always occur in response to evidence of benefit and that it could act as the driver to produce such evidence, we feel this represents an unacceptably high-risk approach to take in the field of health professions education where the acquisition of core disciplinary principles underpins the development of clinical professional competencies. Academic education providers must be mindful of this when deciding on the best ways to achieve educational outcomes in an ecosystem that is expanding to include the field of OOCs.

The high prevalence of MOOCs from the USA and UK may be a result of the exclusion of articles in languages other than English, but this not unusual in the literature [2, 69]. This skew towards developed, Western countries being the implementers and evaluators of MOOCs may impact upon participant perceptions and management of global health needs. This dominance of courses from developed countries is concerning, particularly when MOOCs are presented as educational alternatives for health care professionals in resource-constrained environments and developing countries [70].

While OOCs may be used to facilitate qualitative changes in teaching and learning practice, they require an approach to design that is quite different to the predominant form of MOOC [54]. Five studies in this review reported on the pedagogical framework used to design the course. In three cases the framework described was an xMOOC, the most common form of MOOC currently being implemented by the major providers. Institutions that choose technology platforms like Coursera and Udemy may do so in an attempt to focus on developing content rather than technology, but this means that educators may not have much choice in the kinds of activities their students complete. In about half of the articles the specific activities that participants were required to complete in the courses were not reported and, when they were, included watching videos and answering questions in forum discussions. While there is strong evidence in support of the notion that learning is socially constructed and that interaction is especially important in online learning, few studies in this review included elements that could be described as truly interactive. For example, the use of ‘embedded videos’ or ‘online lessons / modules’ are not interactive, despite author claims. Even in cases where articles in this study demonstrated an innovation in the MOOC space by, for example, including virtual patient cases in the traditional MOOC infrastructure, they still analysed outcomes using server logs and participant satisfaction surveys [39]. xMOOCs are arguably the least pedagogically sound variant if the outcome of interest is a qualitative change in teaching and learning behaviour, and they have been criticised for adopting a knowledge transmission mode of learning. In essence, they are considered to be technology-enriched, traditional, teacher-centred modes of instruction [8]. As this area of practice continues to evolve, clear distinctions between different kinds of MOOCs are becoming increasingly problematic. Future courses will need to integrate approaches across both formats [2]. Such MOOCs may be more likely to enhance innovative teaching and learning practices to inform the established ‘traditional’ method of health professions education. With this in mind, we feel the findings of the present review do not so much represent ‘evidence of a lack of effect’ as they depict ‘a lack of evidence of effect’. The distinction between the two positions is quite overt. The magnitude of interest in this field suggest OOCs may well be a model of education worthy of our attention. The precise nature of its suitability within academic healthcare education providers to address specific learning needs, however, is less clear. The tailoring of different types of OOCs to specific applications within this context will likely be an area of intense interest for future research.

The aim of using economic analyses for educational innovations is to provide low cost and high value approaches to teaching and learning, allowing evidence-based decision-making about the most appropriate allocation of what are often limited resources in an educational context [71, 72]. No such evidence for OOCs emerged from this review. While some economic analyses of MOOCs have previously been conducted, results have been difficult to interpret. For example, Hollands and Tithali (2014) [70] found that, while the cost per learner of some MOOCs may be lower than for traditional online courses, they may only be cost-effective for the most motivated of learners. While the course itself may be costed less than equivalent campus-based courses, such simplistic modelling fails to acknowledge the costs associated with student services such as academic counselling, library services, tutoring, and proctoring for assessment [73, 74]. Inclusion of such factors in MOOC modelling has high potential to render the courses prohibitively expensive [70]. This does not mean that OOCs are unable to offer innovative, low cost, high value avenues for health professions education. However, until economic evaluations of theoretically and pedagogically sound OOCs are conducted, any claims toward these aspirations lack credibility. The combination of making open courses available to vulnerable learner populations, such as those in low income countries, along with fees for certification in the absence of high quality educational evidence of student outcomes and learning experience, further raises concerns of moral and professional accountability [75].

A crucial issue emerging from this review is the lack of strong evidence to support student learning via OOCs. One of the challenges facing research in this field is the question of *how* institutes should use the high volume of data generated from mass participant interactions within a learning environment [76]. Advanced automated analytic processes ( e.g. data mining) may assist such challenges but are scarcely accessible within health professions education. Furthermore, the availability of large data sets of user interactions within online platforms does little to inform health professions educators about the impact of their intervention upon learning and behaviour. Inherent challenges with OOC research such as incomplete databases and distribution across multiple platforms and academic institutions further highlights the need to critically examine the way we conduct research in this space to ensure ‘future proofing’ against the replication of previous pitfalls [6]. In order to improve the quality of data acquisition, it appears essential to develop a collaborative culture among researchers and educators operating within this field. In order for health professions educators to optimise the *value* of data arising from such courses for their disciplines, it would be prudent to establish a minimum standard of research robustness at the course design phase. Based on the stark lack of such high-quality data, it would be reasonable to expect further such studies to significantly impact upon future review conclusions in this area.

### Limitations

An important factor limiting the applicability of our findings to health professions education is the very low level of evidence included within this review – with the largest volume of information coming from descriptive and commentary articles (*n* = 46). Findings should thus be interpreted with due caution in light of this fact. We also added one additional outcome pillar related to the ‘learner experience (opinions / attitudes)’ to the proposed method outlined in our published review registration protocol. This was in response to the nature and amount of data that emerged from several included papers that we felt warranted inclusion. While our framework encapsulated domains we felt to be of principal interest for critical evaluation related to this field of research, this was based upon consensus within our team rather than that of published critical literature. For example, we did not evaluate OOC completion rates, despite being commonly reported, as it was felt to confer minimal relevance of the impact of OOCs for health professions education. Future critical analyses in this field may adopt alternate approaches to ours.

## Conclusion

This review found minimal high-quality evidence that could be used to support decision-making around the inclusion of MOOCs in the field of health professions education. From 2016 to 2019 there has been an increase in the volume of published studies in this domain of practice, albeit with only a small increase in rigour. The majority of articles prior to 2016 included commentary and opinion pieces, while those after 2016 have tended towards descriptive studies that captured simplistic data from participants. While OOCs may turn out to be a disruptive innovation with the potential to influence the nature of the teaching and learning interactions in health professions education, there is currently very limited robust evidence to support the claim. The ability for MOOCs to increase access to education through overcoming geographic boundaries and administrative processes is of significant appeal, however close attention needs to be directed towards comprehensive, multifactorial evaluation of such courses from the perspectives of professionally accountable education institutes. There is an overt need for a vast increase in high quality research in this field. It is our belief that the implementation of MOOCs in health professions education cannot be upheld as sound, evidence-based pedagogical practice until future research demonstrates their precise role and effect on outcomes that are of critical importance to health professions education institutions.

## Additional files

Additional file 1: **Table S1.** Quality appraisal of included studies – reviews (AMSTAR checklist). (DOCX 22 kb)

Additional file 2: **Table S2.** Quality appraisal of included studies – descriptive / case-series studies (The Joanna Briggs Institute). (DOCX 30 kb)

Additional file 3: **Table S3.** Quality appraisal of included studies – randomised controlled trial (Cochrane Risk of Bias Tool). (DOCX 35 kb)

Additional file 4: **Table S4.** Quality appraisal of included studies – narrative, expert opinion, text (Joanna Briggs). (DOCX 30 kb)

Additional file 5: **Table S5.** Quality appraisal of included studies – cohort/case-control studies (The Joanna Briggs Institute). (DOCX 21 kb)

Additional file 6: **Table S6.** Quality appraisal of included studies – qualitative studies (The Joanna Briggs Institute). (DOCX 21 kb)

## Data Availability

All relevant data collected during this study will be uploaded and shared on the publicly available repository at the University of the Western Cape (http://repository.uwc.ac.za/), when the the final article is published.

## Abbreviations

AMSTAR: A MeaSurement Tool to Assess systematic Reviews
CEBM: Centre for Evidence Based Medicine
CINAHL: Cumulative Index to Nursing and Allied Health Literature
DOCC: Distributed Online Collaborative Course
MOOC: Massive Open Online Course
NHMRC: National Health and Medical Research Council
OOC: Open Online Course
PROSPERO: Prospective Register of Systematic Reviews
RCT: Randomised Controlled Trial
SPOC: Small Private Online Course
