# Supplementary material for: These may not be the courses you are seeking: a systematic review of open online courses in health professions education
Source: BMC Med Educ. 2019 Sep 14;19:356. doi: 10.1186/s12909-019-1774-9 (PMC6744630; doi:10.1186/s12909-019-1774-9)
Supplement: Supplementary file 1 — Table S1. Quality appraisal of included studies – reviews (AMSTAR checklist). (DOCX 22 kb) [file 12909_2019_1774_MOESM1_ESM.docx]

Table S1. Quality appraisal of included studies – reviews (AMSTAR checklist).

|  | Inacio  2015 [32] | Liyanagunawardena  2014 [3] | Subhi  2014 [59] | Zhao  2018 [65] |
| --- | --- | --- | --- | --- |
| Was an 'a priori' design provided? | Yes | No | Yes | No |
| Was there duplicate study selection and data extraction? | Unclear | Unclear | Yes | Yes |
| Was a comprehensive literature search performed? | Unclear | Yes | Unclear | Yes |
| Was the status of publication (i.e. grey literature) used as an inclusion criterion? | Yes | Yes | Yes | No |
| Was a list of studies (included and excluded) provided? | Yes | Yes | No | No |
| Were the characteristics of the included studies provided? | Unclear | Yes | Yes | No |
| Was the scientific quality of the included studies assessed and documented? | No | Unclear | No | Yes |
| Was the scientific quality of the included studies used appropriately in formulating conclusions? | No | Unclear | No | No |
| Were the methods used to combine the findings of studies appropriate? | No | Yes | Yes | Yes |
| Was the likelihood of publication bias assessed? | No | No | No | No |
| Was the conflict of interest included? | No | No | No | No |
