# Supplementary material for: These may not be the courses you are seeking: a systematic review of open online courses in health professions education
Source: BMC Med Educ. 2019 Sep 14;19:356. doi: 10.1186/s12909-019-1774-9 (PMC6744630; doi:10.1186/s12909-019-1774-9)
Supplement: Supplementary file 3 — Table S3. Quality appraisal of included studies – randomised controlled trial (Cochrane Risk of Bias Tool). (DOCX 35 kb) [file 12909_2019_1774_MOESM3_ESM.docx]

Table S3. Quality appraisal of included studies – randomised controlled trial (Cochrane Risk of Bias Tool).

| Domain | Hossain 2015  [30] |
| --- | --- |
| Random sequence generation | Low risk |
| Allocation concealment | Low risk |
| Blinding of participants | High risk |
| Blinding of outcome assessment | Low risk |
| Incomplete outcome data addressed | Low risk |
| Selective reporting | Low risk |
