# Supplementary material for: These may not be the courses you are seeking: a systematic review of open online courses in health professions education
Source: BMC Med Educ. 2019 Sep 14;19:356. doi: 10.1186/s12909-019-1774-9 (PMC6744630; doi:10.1186/s12909-019-1774-9)
Supplement: Supplementary file 4 — Table S4. Quality appraisal of included studies – narrative, expert opinion, text (Joanna Briggs). (DOCX 30 kb) [file 12909_2019_1774_MOESM4_ESM.docx]

Table S4. Quality appraisal of included studies – descriptive / case-series studies (The Joanna Briggs Institute).

|  | Random sample | Criteria defined | Confounding factors identified + strategies | Objective outcomes | Descriptions of groups | Follow up sufficient | Outcome of withdrawn participants reported | Outcomes measured reliably | Appropriate statistical analysis |
| --- | --- | --- | --- | --- | --- | --- | --- | --- | --- |
| Evans 2017 [19] | No | Unclear | No | Unclear | N/A | Yes | No | Unclear | No |
| Fricton 2015 [21] | No | N/A | Yes | Yes | Yes | Yes | Yes | Yes | Yes |
| Gooding 2013 [24] | No | N/A | Unclear | Yes | Yes | Yes | Yes | Unclear | Yes |
| Harvey 2014 [25] | No | N/A | Unclear | Yes | Yes | Yes | Yes | Unclear | Yes |
| Harvey 2017 [26] | Yes | Yes | No | Yes | N/A | Yes | No | Unclear | Yes |
| Hoedebecke 2018 [29] | No | No | Yes | No | No | Unclear | No | Unclear | Yes |
| Jacquet 2018 [33] | No | No | No | Yes | Yes | Yes | No | Unclear | Yes |
| King 2014 [37] | No | N/A | Unclear | Unclear | No | Yes | No | Unclear | Yes |
| Kononowicz 2015 [39] | N/A | N/A | Yes | Yes | No | Unclear | No | Yes | Yes |
| Lan 2019 [40] | No | Yes | Yes | Yes | Yes | Yes | Unclear | Yes | Yes |
| Lunde 2018 [42] | Unclear | No | No | No | N/A | Unclear | No | No | No |
| Magana 2018a [43] | Unclear | Yes | No | Yes | N/A | Yes | No | Yes | Yes |
| Magana 2018b[44] | Unclear | Yes | No | Yes | N/A | Yes | No | Yes | Yes |
| Medina 2017 [48] | Unclear | Yes | No | Yes | N/A | Yes | No | Unclear | Yes |
| Milligan 2014 [49] | No | N/A | Unclear | Unclear | No | Unclear | Unclear | Unclear | Unclear |
| Perez-Moreno 2018 [50] | Unclear | Yes | No | Yes | N/A | Yes | No | Unclear | Yes |
| Robinson 2016 [53] | Unclear | No | No | Yes | N/A | Yes | No | Unclear | Yes |
| Sitzman 2016 [55] | Unclear | Yes | No | Yes | N/A | Yes | No | Unclear | Yes |
| Sneddon 2018 [57] | No | N/A | No | Yes | No | Yes | No | Unclear | Yes |
| Stokes 2015 [58] | No | N/A | Unclear | Unclear | Yes | Yes | Yes | Unclear | Unclear |
| Swinnerton 2017 [60] | Unclear | Yes | Unclear | Yes | N/A | Yes | No | Unclear | Yes |
| Wan 2016 [64] | Unclear | No | No | No | N/A | Yes | No | Unclear | No |

N/A = not applicable
