# Supplementary material for: These may not be the courses you are seeking: a systematic review of open online courses in health professions education
Source: BMC Med Educ. 2019 Sep 14;19:356. doi: 10.1186/s12909-019-1774-9 (PMC6744630; doi:10.1186/s12909-019-1774-9)
Supplement: Supplementary file 5 — Table S5. Quality appraisal of included studies – cohort/case-control studies (The Joanna Briggs Institute). (DOCX 21 kb) [file 12909_2019_1774_MOESM5_ESM.docx]

Table S5. Quality appraisal of included studies – cohort/case-control studies (The Joanna Briggs Institute).

|  | Frank  2016 [20] | Jia  2019 [34] |
| --- | --- | --- |
| Representative sample | Yes | Yes |
| Participants similar point in their training | Yes | N/A |
| Selection bias minimised for both groups | Unclear | Yes |
| Identification and control for confounders | No | Unclear |
| Objectively assessed outcomes | Yes | Yes |
| Follow up sufficient | Yes (no followup) | Yes |
| Outcome of withdrawn participants reported | Unclear | Yes |
| Outcomes measured reliably | Unclear | Yes |
| Appropriate statistical analysis | Yes | Yes |

N/A = not applicable
