# Supplementary material for: These may not be the courses you are seeking: a systematic review of open online courses in health professions education
Source: BMC Med Educ. 2019 Sep 14;19:356. doi: 10.1186/s12909-019-1774-9 (PMC6744630; doi:10.1186/s12909-019-1774-9)
Supplement: Supplementary file 6 — Table S6. Quality appraisal of included studies – qualitative studies (The Joanna Briggs Institute). (DOCX 21 kb) [file 12909_2019_1774_MOESM6_ESM.docx]

Table S6. Quality appraisal of included studies – qualitative studies (The Joanna Briggs Institute).

|  | Rowe  2016 [54] |
| --- | --- |
| Is there congruity between the stated philosophical perspective and the research methodology? | Yes |
| Is there congruity between the research methodology and the research question or objectives? | Yes |
| Is there congruity between the research methodology and the methods used to collect data? | Yes |
| Is there congruity between the research methodology and the representation and analysis of data? | Yes |
| Is there congruity between the research methodology and the interpretation of results? | Yes |
| Is there a statement locating the researcher culturally or theoretically? | No |
| Is the influence of the researcher on the research, and vice-versa, addressed? | Yes |
| Are participants, and their voices, adequately represented? | Yes |
| Is the research ethical according to current criteria or, for recent studies, and is there evidence of ethical approval by an appropriate body? | Yes |
| Do the conclusions drawn in the research report flow from the analysis, or interpretation, of the data? | Yes |
